# A Novel Molecular Analysis Approach in Colorectal Cancer Suggests New Treatment Opportunities

**DOI:** 10.3390/cancers15041104

**Published:** 2023-02-09

**Authors:** Elena López-Camacho, Guillermo Prado-Vázquez, Daniel Martínez-Pérez, María Ferrer-Gómez, Sara Llorente-Armijo, Rocío López-Vacas, Mariana Díaz-Almirón, Angelo Gámez-Pozo, Juan Ángel Fresno Vara, Jaime Feliu, Lucía Trilla-Fuertes

**Affiliations:** 1 Molecular Oncology Lab, La Paz University Hospital-IdiPAZ, Paseo de la Castellana 261, 28046 Madrid, Spain; 2 Biomedica Molecular Medicine SL, C/Faraday 7, 28049 Madrid, Spain; 3 Medical Oncology Service, La Paz University Hospital, Paseo de la Castellana 261, 28046 Madrid, Spain; 4 Biostatistics Unit, La Paz University Hospital-IdiPAZ, Paseo de la Castellana 261, 28046 Madrid, Spain; 5 Biomedical Research Networking Center on Oncology—CIBERONC, Carlos III Healthy Institute ISCIII, 28029 Madrid, Spain; 6 Translational Oncology Group, La Paz University Hospital-IdiPAZ, Paseo de la Castellana 261, 28046 Madrid, Spain; 7 Cátedra UAM-Amgen, Universidad Autónoma de Madrid, Ciudad Universitaria de Cantoblanco, 28049 Madrid, Spain

**Keywords:** colorectal cancer, layer analyses, molecular characterization, immune, personalized therapies

## Abstract

**Simple Summary:**

Colorectal cancer is a heterogeneous disease. Several efforts have been made to characterize this heterogeneity but they have no impact in the clinic. In this work, we used a novel analysis approach based on identifying layers of information using expression data from colorectal tumors and characterized three different layers of information: one layer related to adhesion with prognostic value, one related to immune characteristics, and one related to molecular features. The molecular layer divided colorectal tumors into stem cell, Wnt, metabolic, and extracellular groups. These molecular groups suggested some possible therapeutic targets for each group. Additionally, immune characteristics divided tumors into tumors with high expression of immune and viral mimicry response genes and those with low expression, suggesting immunotherapy and viral mimicry-related therapies as suitable for these immune-high patients.

**Abstract:**

Colorectal cancer (CRC) is a molecular and clinically heterogeneous disease. In 2015, the Colorectal Cancer Subtyping Consortium classified CRC into four consensus molecular subtypes (CMS), but these CMS have had little impact on clinical practice. The purpose of this study is to deepen the molecular characterization of CRC. A novel approach, based on probabilistic graphical models (PGM) and sparse k-means–consensus cluster layer analyses, was applied in order to functionally characterize CRC tumors. First, PGM was used to functionally characterize CRC, and then sparse k-means–consensus cluster was used to explore layers of biological information and establish classifications. To this aim, gene expression and clinical data of 805 CRC samples from three databases were analyzed. Three different layers based on biological features were identified: adhesion, immune, and molecular. The adhesion layer divided patients into high and low adhesion groups, with prognostic value. The immune layer divided patients into immune-high and immune-low groups, according to the expression of immune-related genes. The molecular layer established four molecular groups related to stem cells, metabolism, the Wnt signaling pathway, and extracellular functions. Immune-high patients, with higher expression of immune-related genes and genes involved in the viral mimicry response, may benefit from immunotherapy and viral mimicry-related therapies. Additionally, several possible therapeutic targets have been identified in each molecular group. Therefore, this improved CRC classification could be useful in searching for new therapeutic targets and specific therapeutic strategies in CRC disease.

## 1. Introduction

Colorectal cancer (CRC) has been identified as the most prevalent tumor type. According to GLOBOCAN, there were 19.3 million new cases and 10 million cancer deaths worldwide in 2020. CRC ranks third in terms of incidence, representing 10% of new cancer cases, but the second in terms of mortality, with 940,000 estimated deaths [1]. CRC is a molecularly heterogenic disease, in which molecular alterations influence the growth and survival of tumor cells, as well as their differentiation, apoptosis, and distant metastasis [2]. The heterogeneity presented by this cancer has also been related to the anatomical location of the tumor, since the proximal and distal colon have different embryological origins [3]. In this context, Bufill et al. established the first classification of colorectal cancer, defining two groups: group I or proximal if the tumor was located on the right side, and group II or distal when located on the left side [4]. The American Joint Committee (AJCC) on Cancer Tumor-Node-Metastasis (TNM) staging system is the most common staging system in clinical settings. However, a detailed analysis of the prognostic significance of the 8th edition TNM classification for CRC tumors showed that this staging system is not sufficiently accurate in evaluating the prognosis of CRC in the clinic [5].

Until 2015, different genetic classifications for CRC had been proposed. In that year, the international CRC Subtyping Consortium (CRCSC) reached a consensus on the molecular genetic expression subtyping of CRC using a pooled molecular genetic analysis of 4151 colon tumors. Four colon cancer consensus molecular subtypes (CMS) were identified: CMS1 (microsatellite instability immune, 14%), CMS2 (canonical, 37%), CMS3 (metabolic, 13%), and CMS4 (mesenchymal, 23%). However, 13% of the samples could not be classified into any of the four described molecular subtypes [6]. These unclassified tumors could present high intratumor heterogeneity or correspond to an intermediate phenotype, with characteristics belonging to different molecular subtypes [7]. In non-metastatic disease, the poor prognostic value of CMS4 and the relatively favorable prognosis of CMS1 and CMS2 have been established [7]. Moreover, different studies established associations of CMS with treatment outcomes [8,9,10], and their potential for clinical use in predicting both prognosis and response to systemic therapy has been recently evaluated, with encouraging results [6]. The clinical and therapeutic utility of the different molecular classifications has been discussed [11], but, despite the increasing knowledge, treatments based on a molecular subtype are not currently used in clinical decision making [12].

Computational analyses applied to high-dimensional omics data allow a deeper characterization of the molecular and immune features of tumors. Probabilistic graphical models (PGMs) have been previously used to identify differences in biological processes among several tumor types [13,14,15,16,17,18]. Classification methods, such as sparse k-means [19] and consensus cluster (CC) [20], have previously demonstrated their utility in the establishment of tumor and immune subtypes for breast and bladder cancers [13,18].

The main objective of this study is to expand the knowledge about the molecular classification of CRC, according to the different biological realities of the tumor, with the aim to increase the clinical value of the already established molecular groups.

## 2. Material and Methods

### 2.1. Data Search and Curation

Three colorectal tumor gene expression databases (GSE17536, GSE35896, and GSE39582) were analyzed. The resulting database was processed, removing control and duplicated probes. For this purpose, the variance of each probe was calculated and the most variable probe per gene was chosen. In addition, the batch effect due to the combination of independent databases was corrected using the *limma* R package [21]. Information about the CMS group of each sample was downloaded from the Synapse platform [22]. Finally, clinical data from the three databases were collected and unified for further analysis.

### 2.2. Gene Selection and Probabilistic Graphical Model Analysis

First, those genes with a higher standard deviation in their expression across the dataset (standard deviation > 2) were selected to build the PGM, as previously described [18]. The analyses were done using the *grapHD* package [23] and R v3.2.5 [24]. PGMs are undirected acyclic graphs built in two steps: in the first step, the spanning tree that maximizes the likelihood was established, and then a forward search to add edges to build a graph that preserved the decomposability while minimizing the Bayesian Information Criterion (BIC) with the simplest structure was done. The obtained network was split into branches and the most representative function of each branch was established by gene ontology analyses using the DAVID 6.8 webtool [25]. “*Homo sapiens*” was used as a background and categories Biocarta, GO-FAT, and KEGG were selected.

To make comparisons between groups of samples, functional node activities were calculated as previously described [18]. Briefly, the mean expression of all the genes included in one branch related to the main function of this branch was calculated. Differences in functional node activity were assessed by non-parametric tests.

### 2.3. Biological Layer Analyses

Sparse K-means and the consensus cluster algorithm (CC) were used to explore the molecular information of CRC samples, as previously described [26]. Sparse K-means assigns a weight to each gene according to its relevance, explaining the main variability source in the database. Then, using the genes that were selected by sparse K-means, CC was applied to define the optimum number of groups for each case. Once genes relevant to a layer of information were identified, they were removed from the dataset and the analysis was done again with the remaining genes, allowing the identification of different layers of information. Once the information layers were generated, gene ontology analyses were performed for each layer to derive functional information. Sparse K-means was performed using the *sparcl* package [19] and CC was performed using the *Consensus Cluster Plus* package [20] and R v3.2.5 [24].

Then, we used the biological layer information to establish different classifications based on different tumor features. Differential expression patterns among groups were analyzed by Significance Analysis of Microarrays (SAM), defining a false discovery rate (FDR) below 5% [27]. These analyses were carried out using the TM4 Multiexperiment Viewer (MeV) 4.9 software [28].

### 2.4. Statistical Analyses

GraphPad Prism v6 was used for basic statistical analyses. Network visualization was done in Cytoscape software [29]. Differences in node activity were evaluated using the Kruskal–Wallis comparison method and Dunn’s multiple comparison tests. Survival curves were estimated using the Kaplan–Meier method and compared with the log-rank test, using disease-free survival (DFS) as the event. DFS was defined as the time elapsed between surgery and new onset of disease. All *p*-values were two-sided and considered statistically significant below 0.05.

## 3. Results

### 3.1. Pre-Processing of Gene Expression and Clinical Data

Gene expression datasets (GSE17536, GSE35896, and GSE39582) from the Gene Expression Omnibus repository [30] were merged, including probes with expression data in all datasets. After duplicated probes were removed, the batch effect was corrected and a variability filter was applied, and a dataset with 1700 genes and 805 samples was obtained. Here, 177 samples were from GSE17536, 62 samples from GSE35896, and 566 samples from GSE39582.

### 3.2. Patient Characteristics

RNA-seq data from eight hundred and five CRC patients were used in this study. Clinical characteristics of the cohort are summarized in Supplementary Table S1. The median follow-up time was 37 months and 36 relapses occurred.

In total, 132 samples were assigned to CMS1 (16.3%), 315 samples were assigned to CMS2 (39%), 97 samples were assigned to CMS3 (12%), and 188 samples were assigned to CMS4 (23.3%). Thus, 73 samples (9.4%) were not assigned to any CMS.

### 3.3. Functional Characterization

**Figure 1:**
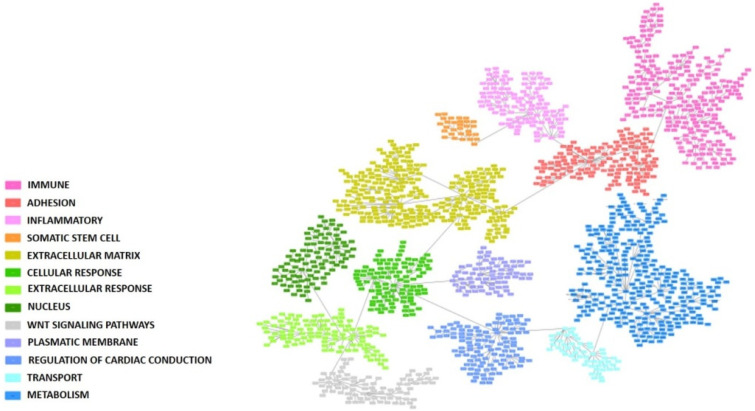
Probabilistic graphical model built using the 1700 more variable genes in the colorectal cancer cohort. Each box represents one gene. Functional nodes are highlighted in the PGM.

A PGM was built with the gene expression profiles of the 1700 more variable genes. Seeking functional structures, 13 functional nodes with an overrepresented biological function were defined: immune, adhesion, inflammatory response, somatic stem cell, extracellular matrix, cellular response, extracellular response, nucleus, Wnt signaling pathway, plasmatic membrane, regulation of cardiac conduction, transport, and metabolism (Figure 1 and Supplementary Table S2).

### 3.4. Biological Layer Analysis

The sparse K-means–CC workflow analyses identified nine biological layers. Each layer was split into two groups, with the exception of the second one, whose optimal classification was found to be in three groups. Each layer’s main function was characterized through gene ontology (Table 1). Layers 1 and 5 were related to adhesion processes, layers 2 and 6 were related to metabolic pathways, and layers 3 and 8 were related to the immune response. The two adhesion layers and the two immune ones were equivalent, respectively, dividing CRC patients into similar groups (Supplementary Figure S1). Therefore, three main biological layers of information were established, an adhesion layer, an immune layer, and a molecular layer, the last one grouping all the information provided by metabolic, extracellular, and digestion classifications.

### 3.5. Adhesion Layer

**Figure 2:**
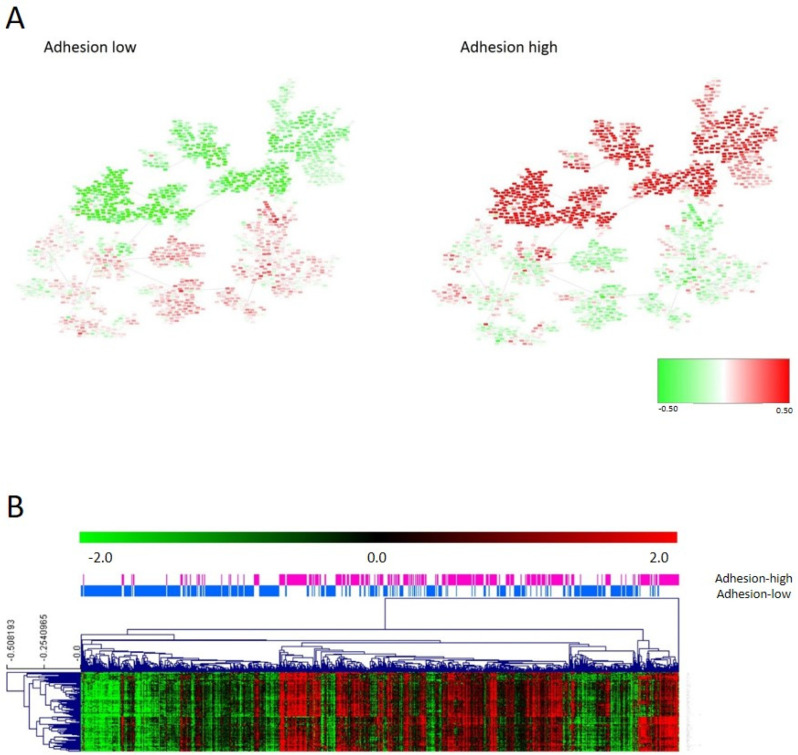
(**A**) Heatmap of the mean gene expression of adhesion groups in the PGM. Red = overexpressed. Green = underexpressed. (**B**) One hundred differentially expressed genes in the adhesion layer between adhesion 1 and 2 groups identified by SAM.

**Figure 3:**
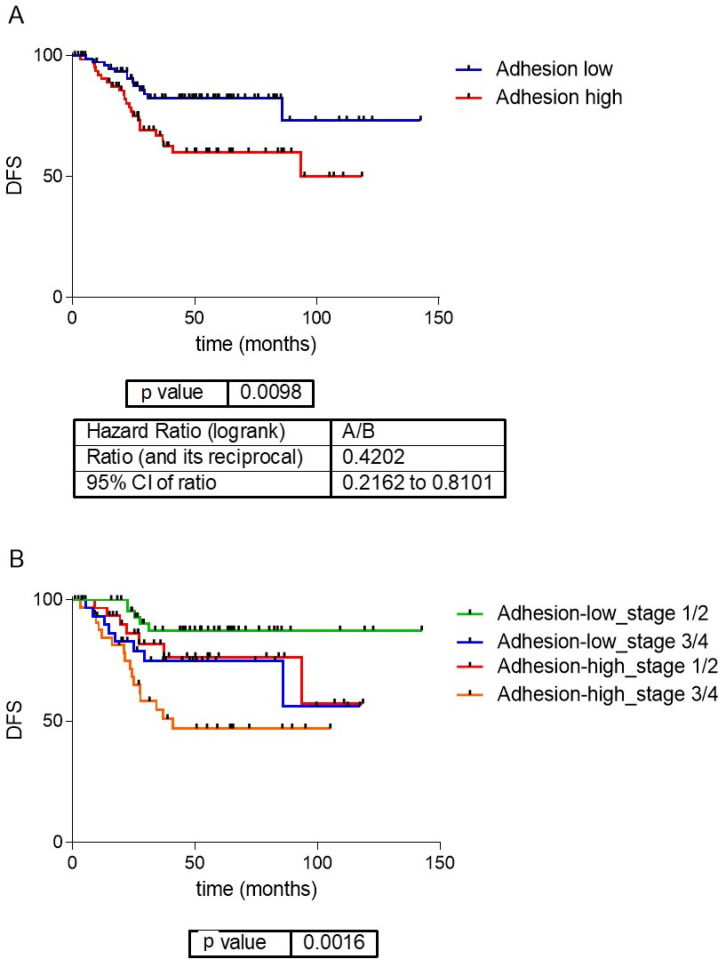
(**A**) Survival analysis of the adhesion groups. Low-adhesion tumors showed a significantly better prognosis than high-adhesion ones. (**B**) Survival curves of the adhesion groups by tumor stage. DFS: disease-free survival.

The first and fifth layers were related to cellular adhesion and divided the samples in a redundant way across the database, so both layers were merged into the adhesion layer. Genes included in the adhesion layer are shown in Supplementary Table S3. CC determined that samples should be divided by their adhesion features into two different groups: adhesion 1, including 454 samples, with lower expression of the adhesion genes, and adhesion 2, including 351 samples, with higher expression of the adhesion genes (Figure 2A). Differentially expressed genes between adhesion groups were identified using SAM and were mainly codified for proteins located in the extracellular matrix, such as collagens, and were related with adhesion functions (Figure 2B and Supplementary Table S4). Patients with low-adhesion tumors had a better prognosis (*p* = 0.0098, HR = 0.42, 95%CI = (0.21–0.81)) (Figure 3). Additionally, there were differences in DFS combining the information of the tumor stage and adhesion groups, with adhesion-high stage 3/4 tumors having significantly worse prognosis and adhesion-low stage 3/4 tumors being comparable in prognosis to adhesion-high stage 1/2 tumors (Figure 3B). No differences in the distribution in each adhesion group according to tumor location and stage were found.

In order to perform a deeper characterization, differences between high- and low-adhesion groups were evaluated by functional node activity, as defined by the PGM (Supplementary Figure S2). The high-adhesion group had higher activity of adhesion, immune response, inflammatory response, stem cell, and extracellular matrix functional nodes. Meanwhile, the low-adhesion group presented higher activity of Wnt signaling pathway, plasmatic membrane, transport, metabolism, and regulation of cardiac conduction functional nodes.

### 3.6. Immune Layer

**Figure 4:**
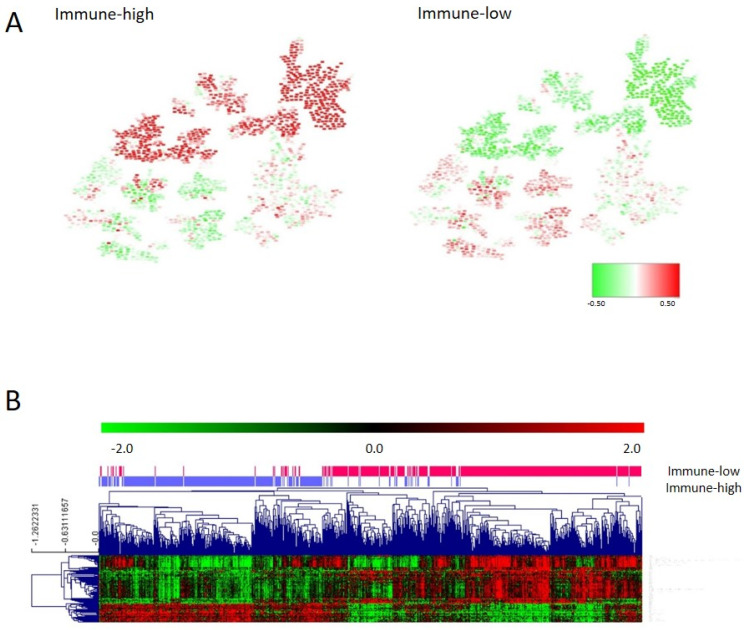
(**A**) Heatmap of the mean gene expression of immune groups in the PGM. Red = overexpressed. Green = underexpressed. (**B**) One hundred differential genes between immune groups identified by SAM.

As for the adhesion layer, the final immune layer was built merging genes from the third and eighth layers (immune response and inflammatory response) (Supplementary Table S3). The CC determined that the immune layer should be divided into two groups: immune 1, renamed as the immune-high group, with 364 tumors showing higher expression of genes related to the immune response, and immune 2, renamed as the immune-low group, including 441 tumors with lower expression of these genes. Differences in gene expression between both groups were evaluated using SAM (Figure 4A). Most of the differential genes belonged to the human leukocyte antigen (HLA) complex gene family (Supplementary Table S4). The immune layer had no prognostic value in our series (*p* = 0.57, HR = 0.82, 95%CI = (0.42–1.59)) (Supplementary Figure S3). No differences according to the distribution of tumor location and stage in each immune group were found.

Functional node activity analysis showed that tumors in the immune-high group had higher expression of genes in the immune, inflammatory, cellular adhesion, and extracellular matrix functional nodes. On the contrary, immune-low tumors had higher expression of genes located in the transport, Wnt signaling pathway, nucleus, and regulation of cardiac conduction functional nodes (Figure 4, Supplementary Figure S4, and Supplementary Table S4).

**Figure 5:**
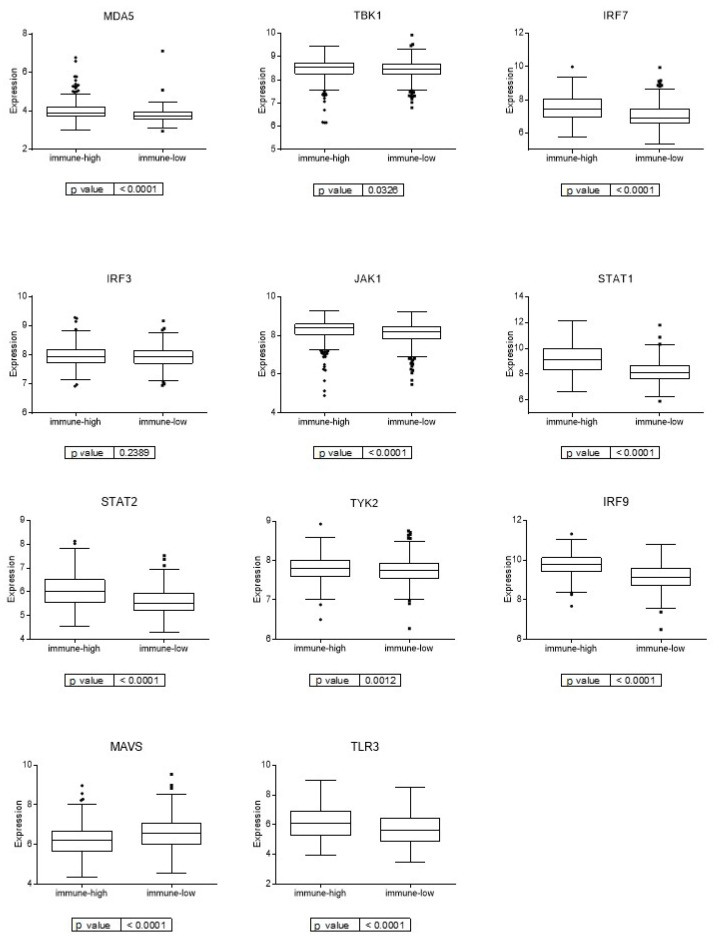
Expression of genes involved in viral mimicry response in the two immune groups.

As the viral mimicry response has gained relevance in the last few years in cancer related to immune response activation [31], we studied the expression of the genes involved in the viral mimicry response in the two immune groups. These immune groups presented differential expression of the genes involved in the viral mimicry response, being higher in the immune-high group (Figure 5).

### 3.7. Molecular Layer

**Figure 6:**
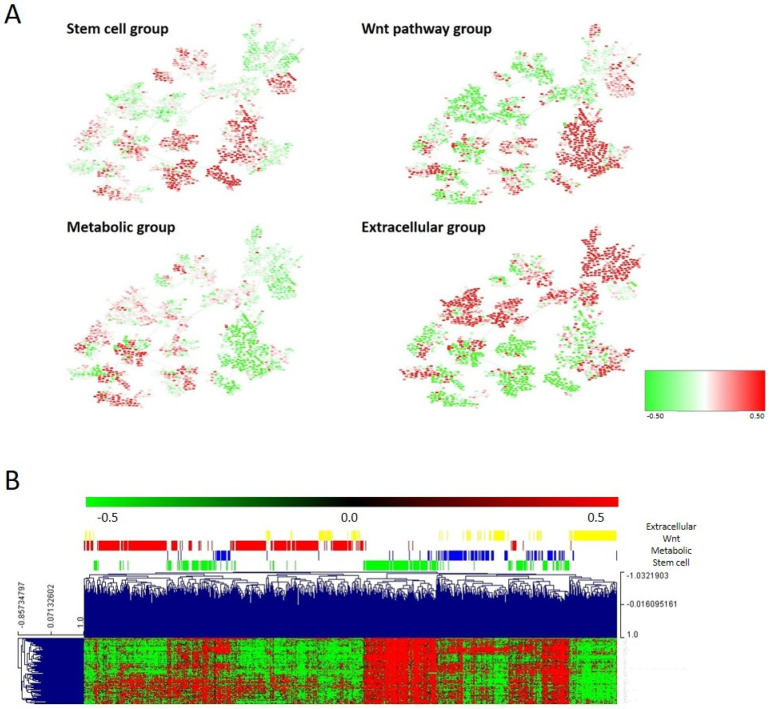
(**A**) Heatmap of the mean gene expression of the four molecular groups in the PGM. Red = overexpressed. Green = underexpressed. (**B**) One hundred differential genes between the four molecular groups.

The molecular layer resulted from merging the second, fourth, and sixth layers and it was divided into four groups by CC. Differences between molecular groups were evaluated using the PGM and the node activities. These analyses showed that molecular group 1 had higher activity in stem cell, nucleus, regulation of cardiac conduction, and transport nodes; molecular group 2 had higher activity of metabolism nodes; molecular group 3 had higher activity of nucleus and Wnt signaling pathway nodes; and molecular group 4 had higher activity of cell adhesion, extracellular matrix, and extracellular response nodes. Therefore, molecular group 1 (221 tumors, 27%), which presented the highest activity for the stem cell functional node, was designated as the stem cell group. Molecular group 2 (137 tumors, 17%), which had the highest activity of metabolism nodes, was named the metabolic group. Molecular group 3 (300 tumors, 37%), which had the highest activity of Wnt signaling pathway nodes, was named the Wnt pathway group. Finally, molecular group 4 (147 tumors, 18%), which had the highest activity of extracellular response and extracellular matrix nodes, was named the extracellular group (Figure 6A, Supplementary Figure S5).

The stem cell node contained genes involved in stem cell maintenance, such as VANGL2 or PBX1. The metabolic node was formed by genes directly involved in metabolism, such as PHGD or PSAT1, and other genes, such as CTSE or REG4. The Wnt pathway node contained genes involved in the Wnt signaling pathway: RNF43, DKK4, LRP4, AXIN2, etc. The extracellular matrix node was mainly formed by collagens. Regarding the association of the defined molecular groups with clinical parameters, the tumor location was distributed significantly different between molecular subtypes (*p* < 0.0001), with the stem cell and Wnt groups mainly composed of distal tumors and the metabolic and extracellular groups by proximal tumors. No differences regarding the distribution of tumor stages across molecular groups were found.

Differentially expressed genes between these molecular groups were identified using SAM (Figure 6B and Supplementary Table S4). The stem cell group showed also the overexpression of genes related to fatty acid metabolism, such as UGT1A1 or UGT1A5, and mucins. The metabolic group showed the overexpression of genes involved in metabolic pathways, including cholesterol or tryptophan metabolism. The Wnt group showed also the overexpression of genes involved in retinol metabolism or epidermal growth factor receptor binding, among others. The extracellular group showed the overexpression of plasma membrane genes.

The survival analysis of these four groups showed no significant differences; however, the extracellular group had a better prognosis than the other three groups (*p* = 0.0086, HR = 0.41 95%CI: (0.13–0.73)) (Supplementary Figure S6).

### 3.8. Comparison between Layer Classification and CMS

**Figure 7:**
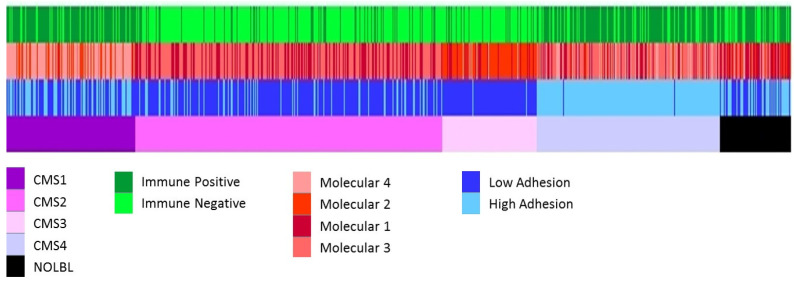
All the classifications of CRC tumors. From top to bottom, immune layer, molecular layer, adhesion layer, and CMS classification groups.

Once we obtained three independent classifications, we compared them with the CMS. Patients belonging to CMS1 and CMS4 were mostly immune-high, whereas CMS2 and CMS3 patients were mostly immune-low. The adhesion layer divided the CMS1 patients by half and most of the CMS4 patients were included in the high-adhesion group, whereas CMS2 and CMS3 were included in the low-adhesion group. According to the molecular layer, most of the CMS1 patients belonged to the extracellular molecular subtype, the CMS2 to the Wnt pathway, and the CMS3 to the metabolic group (Figure 7, Supplementary Table S5).

## 4. Discussion

Colorectal cancer is a molecularly and clinically heterogeneous disease with high rates of incidence and mortality [1]. Both CRC incidence and mortality are expected to increase in the coming years [32]. Therefore, it is essential to explore new molecular markers and therapeutic applications to improve the prognosis and clinical management of this type of tumor. In order to solve the problems derived from the heterogeneity of colorectal cancer, the international Colorectal Cancer Subtyping Consortium (CRCSC) was created, where colon cancer was classified into four consensus molecular subtypes (CMS) [7]. The prognostic value of CMS classification has been proven in metastatic CRC [33,34,35] and recent meta-analysis studies found that the prognostic and predictive value of the CMS is robust [6], but, at the present time, CMS classification has no direct impact on clinical decision making [12]. Many studies have tried to improve the CMS classification for more refined prognosis predictions [36,37,38,39]. However, the discovery of new CRC patient stratification methods is still necessary for the enhanced diagnosis of CRC, screening for novel therapeutic targets, and improved prognostic tools for CRC.

PGMs have demonstrated their utility in the analysis of tumor omics data, being able to structure molecular information from a functional point of view [13,14,40]. Additionally, in other tumor types, such as bladder cancer, sparse K-means–CC analysis provides independent layers of information from the molecular characteristics of the tumor—for instance, immune information [13,18,26]. Therefore, the generation of a classification using this novel approach based on the existence of different informative layers could help to translate into clinical practice the molecular information generated in the context of CRCSC. This approach allows the identification of three different levels of information: the adhesion layer, the immune layer, and the molecular layer.

The adhesion layer has been divided into two groups, high and low adhesion, and has prognostic value, with the group of patients with low adhesion having the best prognosis. Distant CRC metastatic tumor formation is considered to be strongly influenced by the stable adhesion of cancer cells to the small blood vessel walls [41]. In recent years, several studies have shown that adhesion molecules are responsible for tumor progression and metastasis in colorectal cancer; however, the prognostic significance of these markers remains controversial [42]. Moreover, other adhesion proteins, such as FLRT2 and AMIGO2, overexpressed in the high-adhesion group and located in the adhesion and extracellular matrix nodes, respectively, have been suggested to be useful biomarkers for the long-term prognosis of CRC patients [43,44]. As adhesion genes, extracellular matrix genes were also overexpressed in the high-adhesion group. One of these genes was collagen triple helix repeat containing 1 (CTHRC1), related to an increase in cell migration, motility, and invasion. CTHRC1 overexpression was related to poor prognosis in CRC patients and has been defined as a potential diagnostic and prognostic biomarker for patients with CRC [45,46].

Immunotherapy relies on harnessing the body’s immune system to kill cancer cells [47] and it has revolutionized the treatment of several cancers. Immunotherapy has also shown impressive results in the context of CRC. Patients with mismatch repair (dMMR)/microsatellite-instability-high (MSI-H) metastatic CRC have been observed to obtain a prolonged benefit from immune checkpoint inhibitors. Consequently, pembrolizumab and nivolumab +/− ipilimumab have obtained Food and Drug Administration approval for MSI-H/dMMR metastatic CRC [48,49,50]. The immune layer divides CRC tumors into two groups, immune-high and immune-low. A classification capable of identifying immune-related differences, independent of molecular subtype, may identify tumors that will be good responders to immunotherapy. HLA complex genes were overexpressed in the immune-high group. Tumor cells may escape T cell attack through HLA downregulation [51], so the overexpression of HLA complex genes matches with the consideration of this group of patients as optimal candidates for immunotherapy.

The immune-high group had also overexpression of genes involved in the viral mimicry response. The viral mimicry response is a cellular state of active viral response triggered by endogenous stimuli instead of viral infection, in the case of cancer, triggered by retrotransposons [31]. Viral mimicry interprets these retrotransposons as a viral infection and activates the interferon response. The viral mimicry response also increases the adaptive immune response through the increased expression of antigen-processing components and increased expression of retrotransposon-derived peptides [52]. Several of the immune genes on which the immune classification was based were involved in the antigen-processing and presentation process, and they may be the cause of the activation of the viral mimicry response. Moreover, cytidine analogues, azacytidine or decitabine, at low doses, have demonstrated anti-tumor efficacy in colorectal cancer cells by inducing viral mimicry [53], and also it has been demonstrated that they enhance the response to immune checkpoint inhibitors [54]. Therefore, a combination of viral mimicry-related drugs and immunotherapy could be an option for immune-high patients.

The molecular layer has been divided into four groups: stem cells, Wnt pathway, metabolic, and extracellular. Cancer stem cells (CSCs) can regulate cancer invasion, distant metastases, and therapy resistance in CRC, as well as contribute to cancer recurrence in patients [55]. The stem cell subtype presented high expression of genes such as VANGL2 or PBX1, whose function is related to stem cell maintenance. Therefore, these biomarkers could be a possible avenue of study, since colorectal cancer stem cells differ from normal stem cells in their tumorigenic potential and susceptibility to chemotherapeutic drugs [56], which would explain the high percentage of relapses in patients with this type of cancer.

The Wnt signaling pathway plays an important role in the pathogenesis of CRC [57]. Tumors of the Wnt molecular subtype presented high expression of genes such as RNF43, related to alterations in the Wnt signaling pathway [58]. RNF43 encodes an E3 ubiquitin ligase that negatively regulates Wnt signaling, and it is mutated in more than 18% of colorectal adenocarcinomas and endometrial carcinomas. Mutations in *RNF43* have clinical relevance because they implicate novel therapeutic options in CRC. Preclinical studies have shown that mutations in *RNF43* make Wnt-induced cancer cells susceptible to the pharmacological inhibition of Wnt signaling by porcupine. Porcupine is an O-acetyltransferase that is part of the Wnt pathway, and could be postulated as a possible therapy in this type of Wnt-induced tumor [59,60,61]. To date, five porcupine inhibitors have entered phase I/II clinical trials in patients with advanced solid tumors and showed promising preliminary clinical data [62,63]. Porcupine inhibitors were also well tolerated when they were used in combination with anti-PD-1 therapy [64]

In tumors of the metabolic molecular group, the high expression of genes that play an essential role in cancer-specific metabolic reprogramming, such as PHGDH, has been observed [65]. PHGDH is a metabolic enzyme involved in the serine synthetic pathway and it appears to play a central role in supporting cancer growth and proliferation, so it is a promising drug target for cancer therapy. Different PHGDH inhibitors have been reported, but currently they have not yet led to the development of compounds that can be therapeutically used [65]. Another gene overexpressed in the metabolic group was phosphoserine aminotransferase 1 (PSAT1), a gene related to serine biosynthesis. Certain studies concluded that the overexpression of PSAT1 is significantly associated with resistance to chemotherapy with irinotecan, 5-fluorouracil, and leucovorin, so the inhibition of this gene could prevent patients of this group from developing resistance to chemotherapy [66]. Other genes involved in metabolism, such as REG4 and CTSE, were also overexpressed in the metabolic subtype and they have been previously related to CRC prognosis. Regenerating islet-derived type 4 (REG4) is a member of the calcium-dependent lectin gene superfamily and it was associated with a relatively unfavorable prognosis in various cancers, including CRC [67,68]. Cathepsin E (CTSE) is an adverse prognostic factor for survival among rectal cancer patients receiving chemo-radiotherapy [69]. Cathepsins have been implicated to play a role in the invasion and metastasis of colorectal cancer. Inhibitors targeting some cathepsins, such as S and K, are already in clinical evaluation [70], and inhibition of the Reg4-CD44/CD44ICD pathway has been proposed as a future therapeutic target for colon cancer patients [71]. The use of REG4 and CTSE inhibitors could be a targeted treatment for patients of this molecular group. The overexpression of proteins involved in the tricarboxylic acid cycle and mitochondrial metabolism has been previously associated with resistance to oxaliplatin in colorectal cancer organoids [72], highlighting the relevance of metabolism in these tumors.

The extracellular molecular group was characterized by the high expression of collagens, which could be one of the reasons that it is the subtype with the worst prognosis. Among the extracellular matrix adhesive components, type I collagen is one of the most important factors regulating cancer-related events at different tumorigenesis stages [73] The COL1A1 gene encodes a pro-α1 chain of type I collagen, and it has been demonstrated that is overexpressed in colon cancer and it may be a driving gene for colon cancer progression [74,75]. Different inhibitors and drugs that regulate collagen-biosynthesized processes and collagen distribution arrangement have been described, and preclinical studies on collagen-related therapy have demonstrated encouraging outcomes [76]. Patients of this molecular subtype could be candidates for collagen inhibitor therapies.

We compared our three classifications of CRC tumors with the classification through CMS groups. The CMS classification mixed immunological, histological, and molecular information. The present study has been able to corroborate some of the molecular characteristics defined in the CMS, but it has also been possible to identify two layers of information that are independent of the molecular features of the CRC tumors related to adhesion and the immune status. These different levels of information complemented the molecular characteristics exposed in the CMS, and it has also been possible to add new information that allows patients with different CMS to benefit from the same therapeutic strategy.

For instance, although the CRCSC determined that all CMS1 patients were immune-positive [7], our results suggested that 80% of CMS1 patients were immune-high, while 20% of patients in the CMS1 group had low expression of immune response-related genes. This would mean that these patients are not optimal candidates for immunotherapy. Moreover, most CMS2 and CMS3 tumors share the characteristic of having low expression of immune genes (82% and 81% of patients, respectively), being considered cold tumors that do not respond to immunotherapy [77]. However, our molecular classification showed that 18% of CMS2 patients and 19% of CMS3 patients had a high immune status, so they could be candidates for immunotherapy. Therefore, an analysis based on different biological layers allows the more accurate classification of CRC patients according to their immune status, independently of the CMS group to which they belong. The molecular characterization obtained using the described analysis tools provided complementary information to that of the CMS group classification, which may have important implications for the choice of treatment for each patient, such as immunotherapy.

As for the CMS2 group, CRCSC showed that this group presents close similarity to the classical model of CRC carcinogenesis with activation of the WNT and MYC signaling pathways [7]. Although our molecular classification divided CMS2 patients into the Wnt molecular subtype and the stem cell molecular subtype, both subtypes presented high functional activity of the Wnt signaling pathway-related node and both could benefit from porcupine Wnt pathway inhibition therapy.

As for the CMS3 group, the CRCSC established that only patients in this group could benefit from possible therapies with PHGHD and PSAT1 or other metabolism-related molecules. Overall, 24.7% of CMS3 patients do not correspond to the metabolic molecular subtype, so other therapeutic options should be explored for these patients, since, as seen in this study, they are not characterized by the high expression of genes related to metabolism, even though they have been included in CMS3.

Regarding the CMS4 group, our analysis determined that 74% of CMS4 patients were immune-high. This fact is consistent with the CRCSC classification, which described the relationship between CMS4 patients with the presence of high infiltration of cytotoxic T cells [78] and high expression of immune genes [7,37], so, as with CMS1, these patients could be candidates for immunotherapy [79]. However, in this study, it has been possible to determine that 26% of CMS4 patients are immune-low and therefore would not be good candidates for immunotherapy.

On the other hand, CMS4 is the subtype with the worst prognosis [7]. Regarding the adhesion subtype, 98% of CMS4 patients belong to the high-adhesion group, and this is consistent with the survival analysis that determined that the adhesion layer had prognostic value, showing worse prognosis in high-adhesion tumors.

To summarize, this study allows us to minimize the percentage of patients without a specific treatment, since the layer classification allows the inclusion of information about the immune and adhesion status. Thus, patients of the stem cell molecular subtype and the Wnt molecular subtype could benefit from porcupine inhibition therapy, patients of the metabolic molecular subtype from possible therapies related to REG4 and CTSE, and patients of the extracellular molecular subtype from possible therapies related to COL1A1. On the other hand, the classification of patients by immune subtype, independently of CMS, provides valuable information to select the most suitable patients for immunotherapy treatment and viral mimicry therapies.

The study has some limitations. First, validation of all the obtained classifications in an independent CRC cohort is needed. In addition, validation of the proposed therapeutic strategies for each group in cell cultures, organoids, or murine models should be performed. Moreover, only 12% of the tumors were stage IV, so these were underrepresented, as happened in the CMS study. However, this is the group in which possible molecular targets are most interesting because of their potential therapeutic utility. The study used a retrospective cohort, performed prior to immunotherapy administration, which may have changed the prognosis of some patients, especially those with microsatellite instability. Finally, these groups should be studied in the context of other clinical biomarkers, such as RAS/RAF or microsatellite instability.

## 5. Conclusions

In conclusion, the generation of a classification of colorectal cancer according to the different biological realities of the tumor using probabilistic graphic models and layer analysis allowed the identification of four molecular subtypes of colorectal cancer and established two extra independent classifications based on adhesion and immune features, respectively. These classifications may help researchers and clinicians to search for new therapeutic targets and more specific treatments.

## Figures and Tables

**Table 1:** Biological molecular layers obtained by the sparse K-means–consensus cluster workflow analyses and the number of groups into which CRC patients were divided according to each layer.

Layer	Genes	Number of Groups	Main Gene Ontology
1st	98	2	Cellular adhesion
2nd	53	3	Metabolic pathways
3rd	131	2	Immune response
4th	32	2	Digestion
5th	148	2	Cellular adhesion
6th	92	2	Metabolic pathways
7th	78	2	Extracellular response
8th	89	2	Inflammatory response
9th	88	2	Ion calcium and cellular transport

## Data Availability

All generated data are available as supplementary material.

## Supplementary Materials

The following supporting information can be downloaded at: https://www.mdpi.com/article/10.3390/cancers15041104/s1, Supplementary Table S1: Clinical characteristics of CRC patients. Supplementary Table S2: PGM functional nodes gene list. Supplementary Table S3: Genes that composed each layer. Supplementary Table S4: 100 more differentially expressed genes identified by SAM in each group. Supplementary Table S5: Number of patients and percentage of each consensus molecular subtype (CMS) assigned to immune, adhesion, and molecular layers. Supplementary Figure S1: Classifications obtained from biological layer analysis. Supplementary Figure S2: Boxplots representing the activity of each functional node for the adhesion groups. High-adhesion group (AD+), low-adhesion group (AD−). (* *p* ≤ 0.05, ** *p* ≤ 0.01, *** *p* ≤ 0.001, **** *p* ≤ 0.0001); ns: non-significance (*p* > 0.05). Supplementary Figure S3: Survival analysis of the immune groups. Immune-high and immune-low tumor prognosis was not significantly different. DFS: disease-free survival. Supplementary Figure S4: Boxplots representing the activity of each functional node for the immune groups. Immune-high group (IM+), immune-low group (IM−). (* *p* ≤ 0.05, ** *p* ≤ 0.01, *** *p* ≤ 0.001, **** *p* ≤ 0.0001); ns: non-significance (*p* > 0.05). Supplementary Figure S5: Boxplots representing the activity of each functional node of the four molecular cluster subgroups (M1, M2, M3, and M4). (* *p* ≤ 0.05, ** *p* ≤ 0.01, *** *p* ≤ 0.001, **** *p* ≤ 0.0001); ns: non-significance (*p* > 0.05). Supplementary Figure S6: Survival analyses of the four molecular groups. ns: non-significance. DFS: disease-free survival.
