# Supplementary material for: A Novel Molecular Analysis Approach in Colorectal Cancer Suggests New Treatment Opportunities
Source: Cancers (Basel). 2023 Feb 9;15(4):1104. doi: 10.3390/cancers15041104 (PMC9953902; doi:10.3390/cancers15041104)
Supplement: Supplementary file 1 [file cancers-15-01104-s001.zip › Sup figures.pdf]

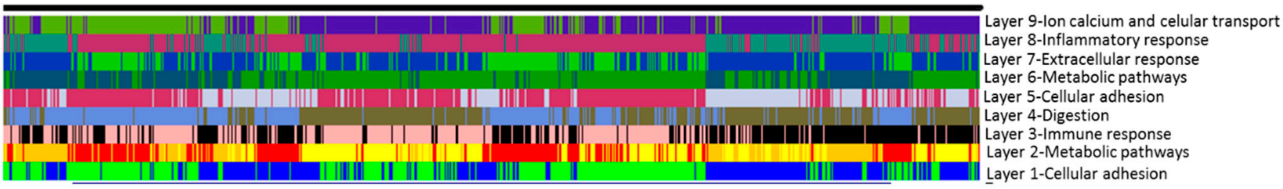

Figure S1: Classifications obtained from biological layer analysis.

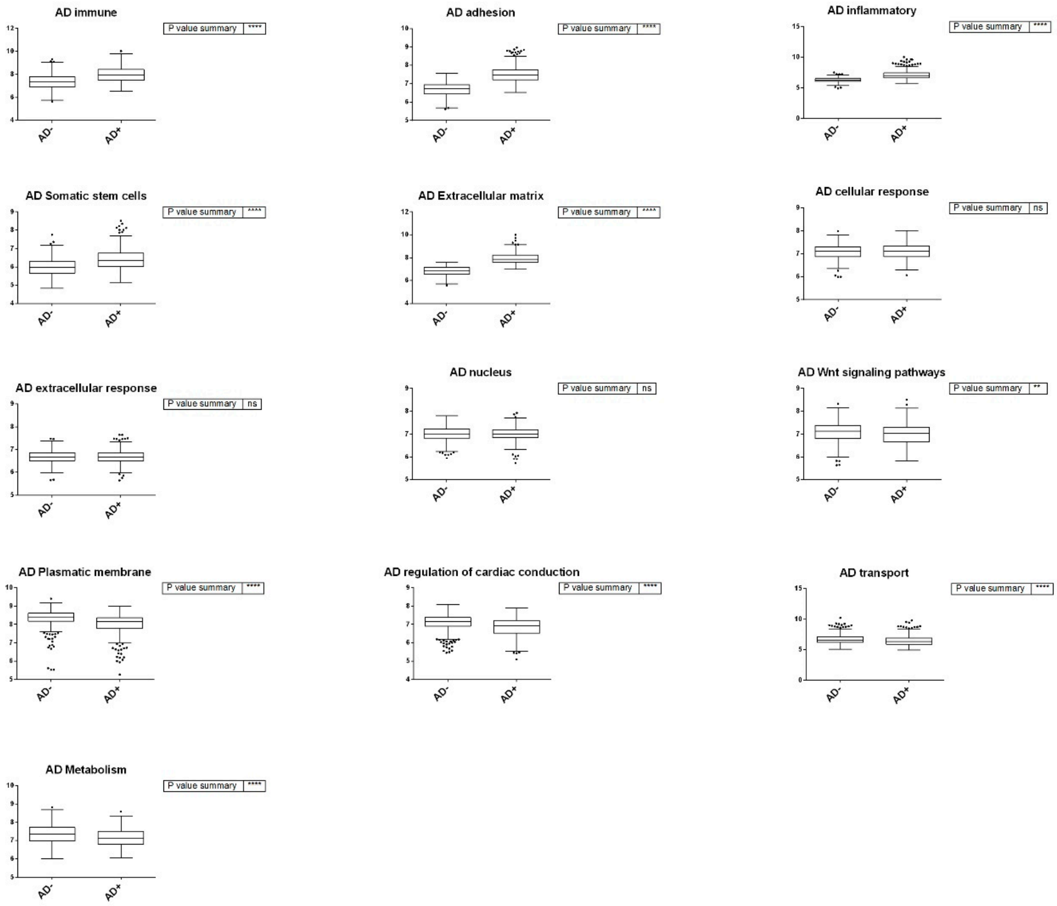

Figure S2: Boxplots representing the activity of each functional node for the adhesion groups. High adhesion group (AD+), low adhesion group (AD-). (*p≤0.05, ** p≤ 0.01, *** p≤0.001, **** p≤0.0001); ns: non significance (p>0.05).

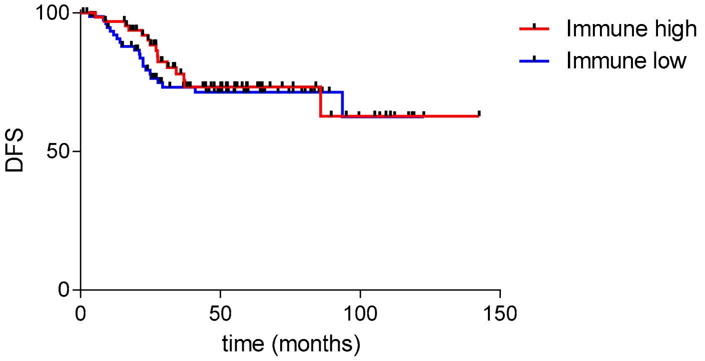

P value	0.5721

Hazard Ratio (logrank)	A/B
Ratio (and its reciprocal)	0.8266
95% CI of ratio	0.4299 to 1.594

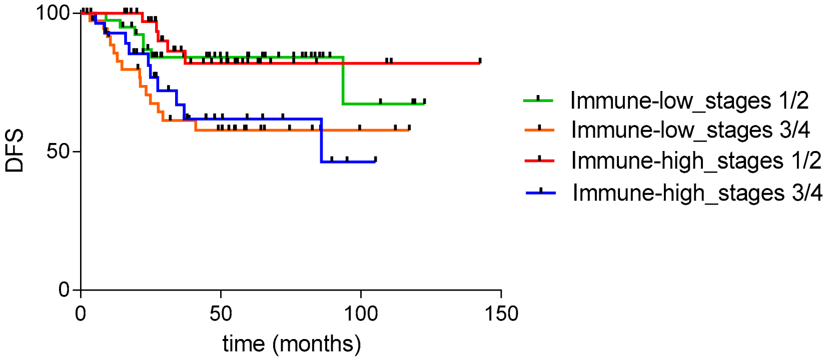

P value	0.0112

Figure S3: Survival analysis of the immune groups. Immune high and immune low tumor prognosis was not significantly different. DFS: disease free survival.

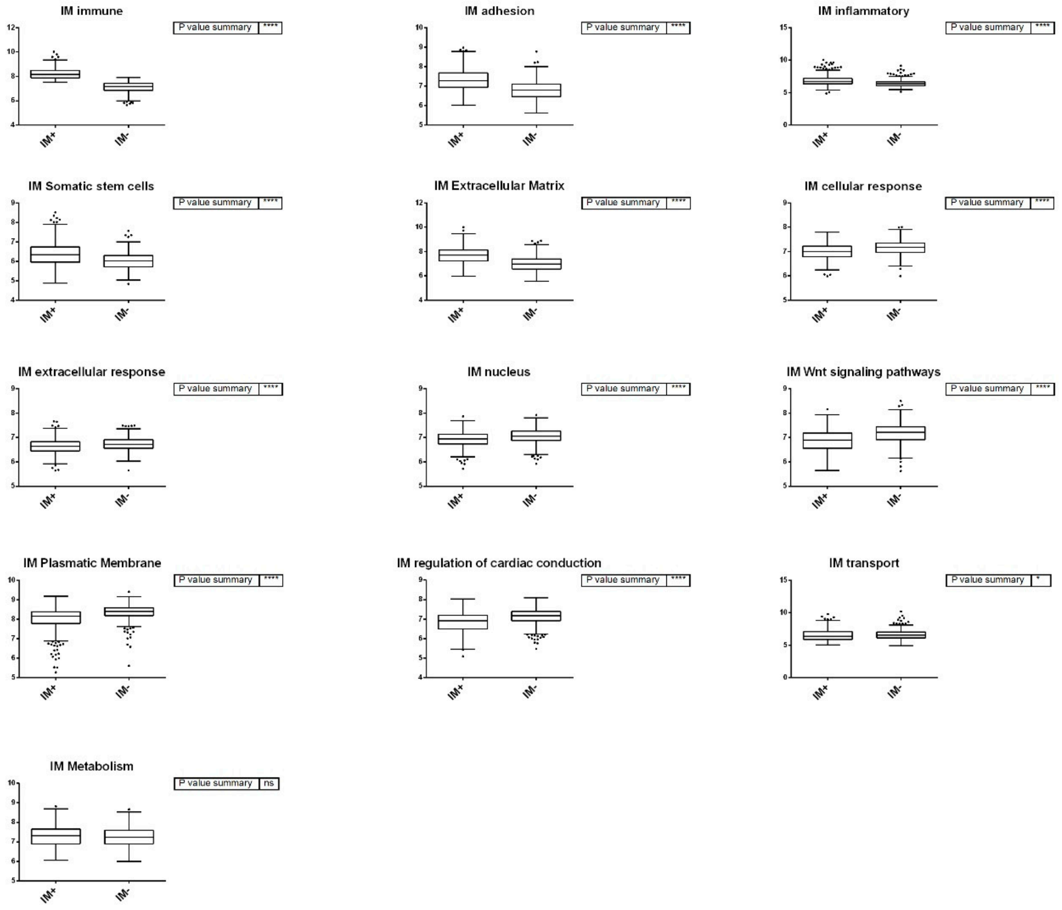

Figure S4: Boxplots representing the activity of each functional node for the immune groups. Immune high group (IM+), immune low group (IM-). (*p≤0.05, ** p≤ 0.01, *** p≤0.001, **** p≤0.0001); ns: non significance (P>0.05).

Node 1 Immune

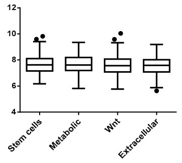

P value	0.7408

Node 2 Cell adhesion

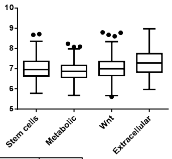

P value	< 0.0001

Stem cells vs. Metabolic	ns
Stem cells vs. Wnt	ns
Stem cells vs. Extracellular	****
Metabolic vs. Wnt	ns
Metabolic vs. Extracellular	****
Wnt vs. Extracellular	***

Node 3 Inflammatory

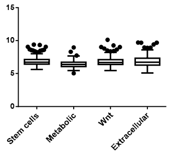

P value	< 0.0001

Stem cells vs. Metabolic	****
Stem cells vs. Wnt	ns
Stem cells vs. Extracellular	ns
Metabolic vs. Wnt	****
Metabolic vs. Extracellular	****
Wnt vs. Extracellular	ns

Node 4 Somatic stem cell

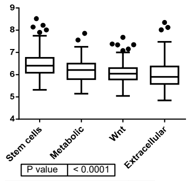

P value	< 0.0001

Stem cells vs. Metabolic	***
Stem cells vs. Wnt	****
Stem cells vs. Extracellular	****
Metabolic vs. Wnt	*
Metabolic vs. Extracellular	**
Wnt vs. Extracellular	ns

Node 5 Extracellular matrix

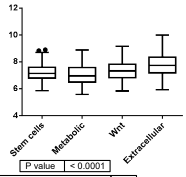

P value	< 0.0001

Stem cells vs. Metabolic	ns
Stem cells vs. Wnt	ns
Stem cells vs. Extracellular	****
Metabolic vs. Wnt	****
Metabolic vs. Extracellular	****
Wnt vs. Extracellular	****

Node 6 Cellular response

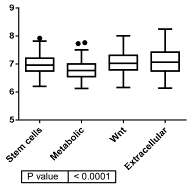

P value	< 0.0001

Stem cells vs. Metabolic	****
Stem cells vs. Wnt	ns
Stem cells vs. Extracellular	ns
Metabolic vs. Wnt	****
Metabolic vs. Extracellular	****
Wnt vs. Extracellular	ns

Node 7 Extracellular response

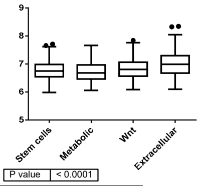

P value	< 0.0001

Stem cells vs. Metabolic	ns
Stem cells vs. Wnt	ns
Stem cells vs. Extracellular	****
Metabolic vs. Wnt	*
Metabolic vs. Extracellular	****
Wnt vs. Extracellular	***

Node 8 Nucleus

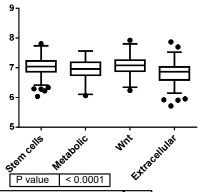

P value	< 0.0001

Stem cells vs. Metabolic	*
Stem cells vs. Wnt	ns
Stem cells vs. Extracellular	****
Metabolic vs. Wnt	***
Metabolic vs. Extracellular	*
Wnt vs. Extracellular	****

Node 9 Wnt signaling pathways

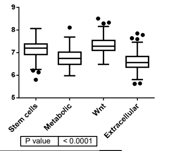

P value	< 0.0001

Stem cells vs. Metabolic	****
Stem cells vs. Wnt	***
Stem cells vs. Extracellular	****
Metabolic vs. Wnt	****
Metabolic vs. Extracellular	ns
Wnt vs. Extracellular	****

Node 10 Plasmatic membrane

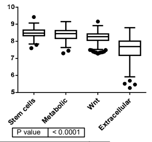

P value	< 0.0001

Stem cells vs. Metabolic	ns
Stem cells vs. Wnt	****
Stem cells vs. Extracellular	****
Metabolic vs. Wnt	****
Metabolic vs. Extracellular	****
Wnt vs. Extracellular	****

Node 11 Regulation of cardiac conduction

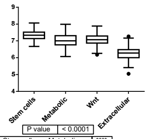

P value	< 0.0001

Stem cells vs. Metabolic	****
Stem cells vs. Wnt	****
Stem cells vs. Extracellular	****
Metabolic vs. Wnt	ns
Metabolic vs. Extracellular	****
Wnt vs. Extracellular	****

Node 12 Transport

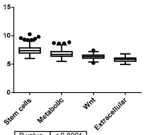

P value	< 0.0001

Stem cells vs. Metabolic	****
Stem cells vs. Wnt	****
Stem cells vs. Extracellular	****
Metabolic vs. Wnt	****
Metabolic vs. Extracellular	****
Wnt vs. Extracellular	****

Node 13 Metabolism

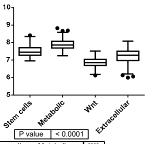

P value	< 0.0001

Stem cells vs. Metabolic	****
Stem cells vs. Wnt	****
Stem cells vs. Extracellular	****
Metabolic vs. Wnt	****
Metabolic vs. Extracellular	****
Wnt vs. Extracellular	****

Figure S5: Boxplots representing the activity of each functional node of the four molecular cluster subgroups (M1, M2, M3 and M4). (*p≤0.05, ** p≤0.01, *** p≤0.001, **** p≤0.0001); ns: non significance (p>0.05).

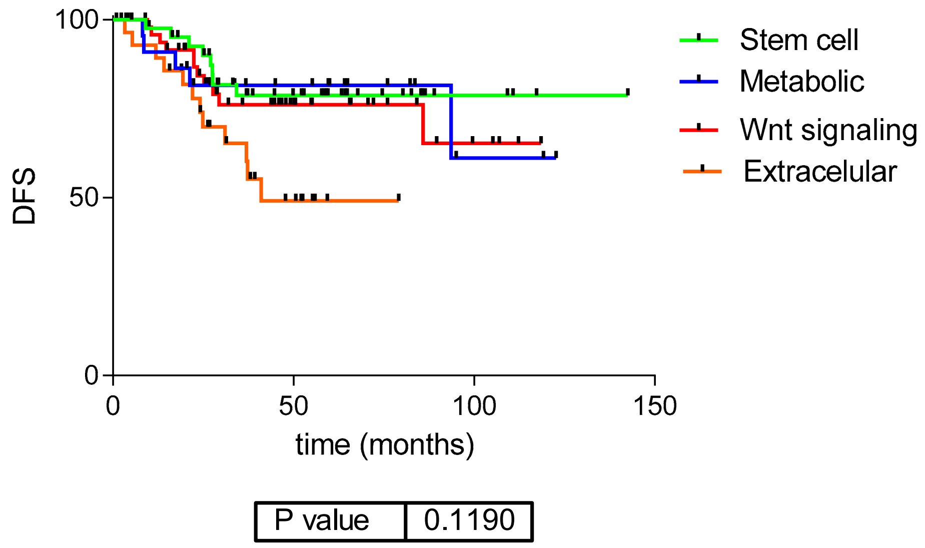

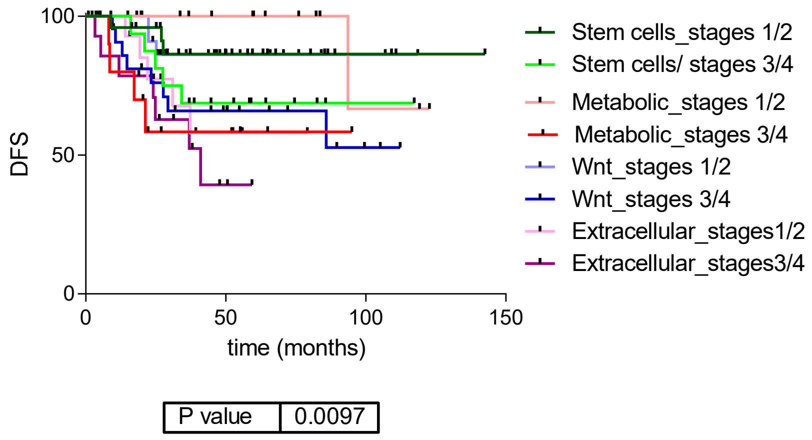

Figure S6: Survival analyses of the four molecular groups. ns: non significance. DFS: disease free survival.
