# Supplementary material for: A Novel Molecular Analysis Approach in Colorectal Cancer Suggests New Treatment Opportunities
Source: Cancers (Basel). 2023 Feb 9;15(4):1104. doi: 10.3390/cancers15041104 (PMC9953902; doi:10.3390/cancers15041104)
Supplement: Supplementary file 1 [file cancers-15-01104-s001.zip › Sup Tables.pdf]

Number of patients	805 (100%)
Age median (range)	66 (26-92)
Gender	
Male	96 (12%)
Female	81 (10%)
Unknown	628 (78%)
Location	
Distal	342 (42%)
Proximal	224 (28%)
Unknown	239 (30%)
8th ed. TNM AJCC Stage	
1	57 (7%)
2	321 (40%)
3	262 (33%)
4	99 (12%)
Unknown	66 (8%)

Table S1: Clinical characteristics of CRC patients.

		CMS1	CMS2	CMS3	CMS4	NOLBL
**ADHESION**	**low adhesion**	68 (52%)	255 (81%)	95 (98%)	2 (2%)	34 (47%)
	**high adhesion**	64 (48%)	60 (19%)	2 (2%)	186 (99%)	39 (53%)
**IMMUNE**	**immune positive**	106 (80%)	57 (18%)	18 (19%)	139 (74%)	44 (60%)
	**immune negative**	26 (20%)	258 (82%)	79 (81%)	49 (26%)	29 (40%)
**MOLECULAR**	**Stem cells**	2 (2%)	123 (39%)	18 (19%)	42 (22%)	36 (49%)
	**Metabolic**	38 (29%)	0 (0%)	73 (75%)	12 (6%)	14 (19%)
	**Wnt pathway**	3 (2%)	191 (60.7%)	0 (0%)	88 (47%)	18 (25%)
	**Extracellular**	89 (67%)	1 (0.3%)	6 (6%)	46 (24%)	5 (7%)

Table S5: Number of patients and percentage of each consensus molecular subtype (CMS) assigned to immune, adhesion, and molecular layers.
